# Hepatitis C virus and HIV infections among people who inject drugs in the Middle East and North Africa: a neglected public health burden?

**DOI:** 10.7448/IAS.18.1.20582

**Published:** 2015-07-28

**Authors:** Ghina R Mumtaz, Helen A Weiss, Laith J Abu-Raddad

**Affiliations:** 1 Infectious Disease Epidemiology Group, Weill Cornell Medical College – Qatar, Cornell University, Qatar Foundation – Education City, Doha, Qatar; 2 Department of Infectious Disease Epidemiology, Faculty of Epidemiology and Population Health, London School of Hygiene and Tropical Medicine, London, United Kingdom; 3 MRC Tropical Epidemiology Group, Department of Infectious Disease Epidemiology, Faculty of Epidemiology and Population Health, London School of Hygiene and Tropical Medicine, London, United Kingdom; 4 Department of Healthcare Policy and Research, Weill Cornell Medical College, Cornell University, New York, NY, USA

## Introduction

People who inject drugs (PWID) are a key population at risk of hepatitis C virus (HCV) and HIV infections. Globally, 63% of PWID are HCV infected [1, 2] and 19% are HIV infected [2], leading to an estimated 10 million and 3 million HCV- and HIV-infected PWID, respectively [1–3]. The Middle East and North Africa (MENA), a region comprising 23 countries from Morocco in the West to Pakistan in the East, is at the centre of major drug production and trade, creating a context of vulnerability to injecting drug use [4]. PWID in MENA are a large, mostly young and stigmatized population experiencing a substantial HCV and HIV burden, with potential for even further HIV epidemic growth. Yet, they lack access to comprehensive and confidential HCV and HIV testing, prevention and treatment services [5].

## A large population at risk

MENA is home to an estimated 626,000 current PWID (range: 335,000–1,635,000) [6], with Iran, Pakistan and Egypt bearing the largest numbers [6]. The population proportion of PWID, at 0.24 per 100 adults, is comparable to global figures [2], but highest in the Eastern part of the region, such as in Iran at 0.43 per 100 adults [6].

## A substantial HCV infection burden

**Figure 1:**
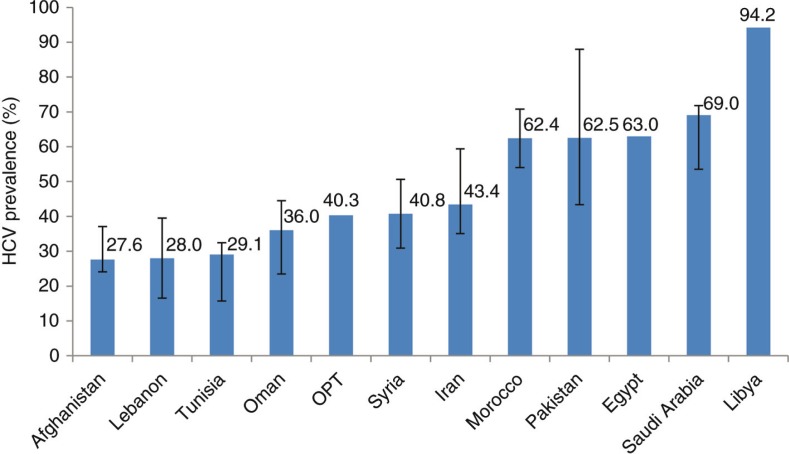
Median HCV prevalence among people who inject drugs in the Middle East and North Africa as per available studies [6]. Error bars represent the lower and upper bounds of the interquartile range if more than one data point was available per country.

Overall, about half of PWID in MENA are HCV infected (median: 44%; interquartile range (IQR): 31–64%), and prevalence as high as 90% has been reported among some PWID populations [6] (Figure 1). In addition to the estimated 300,000 HCV-infected current PWID [6], there could be as many as 2 million HCV-infected people who acquired the infection through past drug injection, but are no longer injecting. In the United States, for example, the number of HCV-infected previous PWID is more than seven times the number of HCV-infected current PWID [7].

The high HCV prevalence and the injecting risk behaviour environment suggest substantial ongoing HCV transmission [6]. This is affirmed by measured and estimated HCV incidence among PWID. In one study in Afghanistan, for example, an HCV incidence rate of 67 per 100 person-years (pyr) has been reported [8]. Preliminary mathematical modelling results suggest that PWID are a major driver of HCV incidence in MENA (Mumtaz *et al*., under preparation).

## Emerging and growing HIV epidemics

Recent evidence has documented HIV epidemics among PWID in one-third of MENA countries [6]. The scale of the epidemic among PWID could be underestimated as the epidemic status remains unknown in half of MENA countries [6]. In some settings, HIV prevalence has reached unprecedented levels, such as in Tripoli, Libya at 87.2% [9]. The common pattern, however, remains that of emerging concentrated epidemics such as in Afghanistan and Egypt [6]. Most epidemics occurred only in the last decade and HIV prevalence hovers around 10–15% [6].

There is also evidence for substantial HIV incidence among PWID. HIV incidence rates of 1.7 [10], 2.2 [8] and 17.2 [11] per 100 pyr have been reported in Pakistan, Afghanistan and Iran, respectively. Modelling work has estimated high incidence in Iran with the majority of infections being due to drug injection [12]. Case notifications also suggest a dominant contribution of PWID to HIV incidence in Afghanistan and Libya [6].

The early phase of the HIV epidemics and the prevalence of risky injecting and sexual practices suggest potential for further HIV epidemic growth among PWID [6]. Recent predictions suggest moderate to high HIV epidemic potential among PWID in countries such as Afghanistan, Egypt, Lebanon, Morocco, Palestine, Saudi Arabia, Syria and Tunisia [13].

## Moving forward

There is an urgent need to prioritize PWID for interventions and to scale up harm reduction services in MENA. In 2014, needle/syringe exchange programmes (NSPs) were implemented in ten MENA countries, and opioid substitution therapy (OST) in six [14]. These do not include Libya and Saudi Arabia, countries with high HCV prevalence among PWID (Figure 1). Among the other countries with substantial HCV infection burden, Morocco is the only one with operational NSP and OST programmes, while in Pakistan and Egypt only NSPs are provided. Iran remains the leader in harm reduction with an NSP coverage of 55–77% among PWID in 2014, and provision of OST through 4200 centres [14]. Limited funding, low and heterogeneous coverage of services, socio-cultural stigma and fear of arrest persist as major barriers for access and provision of harm reduction services [14]. MENA countries could benefit from Iran's experience in implementing harm reduction within the regional social-cultural context. With most PWID starting injecting at a young age, harm reduction should be adapted for young people and linked to other sectors such as education and employment [15].

Alongside prevention interventions, the recent availability of highly effective direct-acting antivirals to treat HCV offers hope for HCV-infected PWID. The prohibitively expensive cost of the drugs remains a major challenge for scale-up. Ensuring affordable access to treatment will only be possible with generic competition or with substantial price reductions on existing or upcoming drugs such as the 99% price discount negotiated by Egypt [16] and a similar discount negotiated recently by Pakistan. Generics are planned to be manufactured within the region, such as in Egypt and Morocco. Generics are being produced in India for as little as $750 for a full treatment course, and production costs may go down to $100 within a few years [17]. As the first Global Health Sector Strategy on Viral Hepatitis is being drafted, concerted efforts are needed for the development of National Strategic Plans for Viral Hepatitis, and possibly Viral Hepatitis Programmes, at country level in MENA, as is already materializing in a few countries including Bahrain, Egypt, Lebanon and Iran. Such programmes can furnish the logistical framework for supporting HCV-related services among PWID through initiatives including testing, treatment and optimally harm reduction, in tandem with National AIDS Control Programme services.

As for HIV treatment, much remains to be accomplished in a region that has one of the lowest antiretroviral therapy (ART) coverages worldwide with a median coverage of 16% (IQR: 6–17%) [18]. Limited HIV testing, the cost of ART to burdened health care systems, and poor access are obstacles for ART uptake and scale-up [19]. The median prevalence of lifetime HIV testing among PWID is 33% (IQR: 16–56%), and is very low in many countries with concentrated HIV epidemics such as in Afghanistan, Pakistan and Egypt [6]. While Voluntary Counselling and Testing (VCT) has been initiated in most countries, uptake of services has been overall weak, partially because of weak non-governmental organizations (NGO) involvement, limited engagement of PWID, and social stigma [5]. Morocco is one exception where the strong civil society has facilitated broad and sizable access to VCT services for different populations [5]. Provision and access to HCV testing is even more limited because of the poor commitment to HCV treatment. Managing the structural barriers of social stigma, poverty, homelessness, criminalization and incarceration will facilitate both HIV and HCV testing, treatment and prevention scale-up for PWID in MENA [20].

## Conclusions

There is a large marginalized population of over half a million PWID in MENA, half of whom are already HCV infected. There is also a larger population of HCV-infected previous injectors who are progressing through the natural course of disease without knowing the status of their infection or the opportunity of treatment. PWID in MENA are also enduring rising HIV epidemics, some of which have already reached high HIV prevalence. Advantage should be taken from the global momentum for tackling viral hepatitis, and courageous decisions are needed at the national level to develop or expand programmes that can tackle HCV and HIV public health burden among PWID. Scale-up of treatment and harm reduction services should be a main pillar of such programmes, alongside innovative strategies to overcome the challenges imposed by social stigma and criminalization.
